# Natural Selection Molds Genomic Insulator Elements

**DOI:** 10.1371/journal.pbio.1001421

**Published:** 2012-11-06

**Authors:** Richard Robinson

**Affiliations:** Freelance Science Writer, Sherborn, Massachusetts, United States of America

**Figure:**
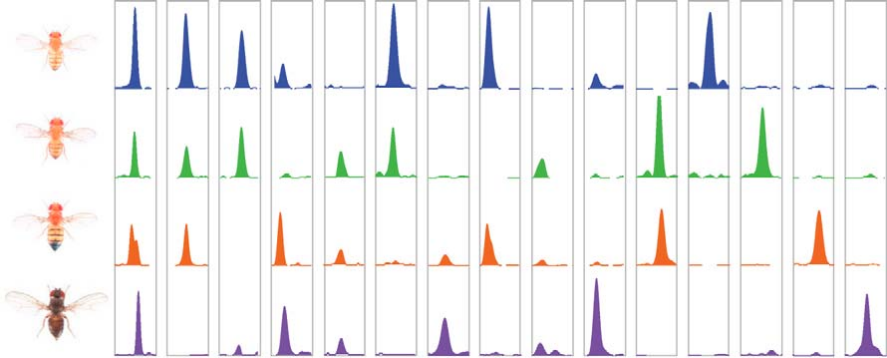
Evolutionary patterns of CTCF binding in the genomes of four species of fruit flies.

The “modern synthesis” of evolutionary biology, first formulated in the early 20th century, combined the ideas of Mendelian genetics and Darwinian evolution to create an outline for evolutionary theory that stressed the central role of genes in evolutionary processes. Of the genetic changes that drive evolution, the earliest to be studied were in proteins with familiar and prominent roles in the cell, such as enzymes or structural proteins.

But since then, the astonishing complexity of genomic regulation has come to be better understood. With that understanding has come the recognition that evolutionary change is often driven by changes in regulatory systems acting behind the scenes, changes that affect when, where, and how much those more familiar genes are expressed. Much of the morphologic diversity within and between species is due these kinds of changes.

Despite their importance, it has been challenging to investigate the exact nature of the processes driving the evolution of specific regulatory elements; for instance, whether observed changes are due to positive evolutionary selection or to neutral drift. In this issue of *PLOS Biology*, Xiaochun Ni, Kevin White, and colleagues tackle this question by examining genome-wide changes in the binding sites for a key gene regulatory protein in multiple species of fruit fly.

CCCTC binding factor (CTCF) is a so-called insulator protein. It binds to DNA to mark the boundaries of large-scale chromosomal regulatory units (such as chromatin domains), preventing unwanted spreading of transcription regulatory signals to adjacent genes. Previous work has shown that the number of CTCF binding sites, and the sequences of those individual sites, has changed over time.

To investigate that nature of that evolutionary change in CTCF binding sites, the authors began by mapping all the binding sites in four species of fly: *Drosophila melanogaster*, the standard laboratory fruit fly; *D. simulans*, which diverged from *D. melanogaster* 2.5 million years ago; *D. yakuba*, which diverged 6 million years ago; and *D. pseudoobscura*, which diverged 25 million years ago. They isolated the binding sites by chromatin immunoprecipitation, in which an antibody to CTCF is used to purify the protein along with its DNA binding sites. They then sequenced these sites and pinpointed their positions in the genomes; this allowed them to determine what sequence changes occurred at equivalent sites among the four species.

They first noticed that, not unexpectedly, the more evolutionary time separating each species from *D. melanogaster*, the further diverged was the set of CTCF binding sites, in both sequence and number. Evolutionary processes were clearly acting on these sites within each species, causing them to change over time. But were these changes an adaptive response to selective pressure, or simply due to random drift in the genetic code?

One piece of evidence favoring selection, the authors found, was that the creation of new sites occurred in each species at a rate far higher than the loss of old sites. In each pair of species where site gain and loss could be inferred, the authors observed that more new sites were created over time than were lost. The authors surmised that this pattern hinted at the work of positive selection for CTCF binding sites.

More rigorous support for the effects of selection came from applying a series of statistical tests to comparisons among older versus newer CTCF sequences. They found that older CTCF sites tended to become stabilized over time, by a process called purifying selection, in which variations away from a given sequence reduce fitness. By contrast, comparing younger CTCF sequences to sequences presumed to evolve neutrally, they found reduced variation within species (polymorphism) in the CTCF sites, evidence of positive selection for the evolution of the binding sites.

Finally, the evolution of new binding sites also correlated with changes in gene expression, in keeping with their gene regulatory role. Moreover, the authors found that among 42 “young” genes essential for fly survival, eight had new CTCF sites nearby that had arisen at about the same time as the gene itself, further supporting a central role for CTCF site creation in fly evolution.

Taken together, these results show that natural selection can and does act on gene regulatory elements, shaping their evolution along with the genes they control. A full understanding of the evolution of the fly will need to take such events into consideration. Similar forces have no doubt been at work in our own lineage, and will be just as important to unravel.

**Ni X, Zhang YE, Nègre N, Chen S, Long M, et al. (2012) Adaptive Evolution and the Birth of CTCF Binding Sites in the** ***Drosophila*** **Genome. doi:10.1371/journal.pbio.1001420**

